# Estrogen receptor-positive breast cancer and adverse outcome in *BRCA2* mutation carriers and young non-carrier patients

**DOI:** 10.1038/s41523-023-00600-8

**Published:** 2023-11-30

**Authors:** Linda Vidarsdottir, Elinborg J. Olafsdottir, Rosa B. Barkardottir, Olöf Bjarnadottir, Jon G. Jonasson, Stefan Sigurdsson, Laufey Tryggvadottir

**Affiliations:** 1 Faculty of Medicine, University of Iceland, Sturlugata 8, Reykjavik, Iceland; 2 BioMedical Center, University of Iceland, Sturlugata 8, Reykjavik, Iceland; 3 Icelandic Cancer Registry, Icelandic Cancer Society, Reykjavik, Iceland; 4 Department of Pathology, Landspitali—University Hospital, Reykjavik, Iceland; 5 Department of Oncology, Landspitali—University Hospital, Reykjavik, Iceland

**Keywords:** Breast cancer, Prognostic markers

## Abstract

Estrogen receptor-positive (ER+) breast cancer generally confers a more favorable prognosis than ER-negative cancer, however, a different picture is emerging for *BRCA2* mutation carriers and young patients. We used nationwide data from population-based registries to study prognostic effects in those two groups. Of all 2817 eligible women diagnosed with breast cancer in Iceland during 1980–2004, 85% had been tested for the Icelandic 999del5 *BRCA2* (c.771_775delTCAAA) founder pathogenic variant. We compared breast cancer-specific survival, effects of ER status, other clinical parameters, and treatment, between three mutually exclusive groups: *BRCA2*-carriers, non-carriers diagnosed 40 years or younger, and older non-carriers. Prevalence of the *BRCA2* mutation among tested patients <=40 years of age was 21.0%, but it was 5.4% among women diagnosed >40 years of age. For ER+ cancer, breast cancer-specific 15-year survival was 49.7%, 55.2%, and 74.7%, among *BRCA2*-carriers, young and older non-carriers, respectively, whereas for ER-negative cancer, survival was similar (64.0–69.3%) for all three groups. Neither *BRCA2* carriers nor young non-carriers did tumor grade 3 predict worse survival than did tumor grade 1. The adverse outcome for the young cases cannot be explained by *BRCA2* mutations, as carriers were excluded from the group. Those two clinically important patient groups need special attention with respect to treatment choices, in particular, if diagnosed with ER+ tumors. It is thus advisable to have knowledge of *BRCA2* status when treatment decisions are made. Finally, it is important to understand the biological basis for the specific nature of ER+ tumors in young women and *BRCA2* carriers.

## Introduction

Breast cancer is a heterogeneous disease and prognosis varies greatly between patient groups. In addition to traditional prognostic factors, biological subtypes defined by gene expression or immunohistochemistry are widely used for treatment decisions^1,2^. Luminal-like breast cancer includes luminal A- and B-like subtypes, characterized by expression of estrogen- (ER) and/or progesterone-receptor (PR). These subtypes confer a more favorable prognosis than triple negative cancer, the best prognosis being observed for the Luminal A-like subtype, which has less proliferative activity than other subtypes. Furthermore, in recent years, multi-gene molecular assays have been increasingly used for making distinctions among patients with luminal disease^3^.

Pathogenic mutations in the *BRCA1* and *BRCA2* genes confer increased lifetime risk of developing breast- and ovarian cancer. The majority of tumors in *BRCA1* mutation carriers are of the triple-negative subtype while over 70% of tumors among *BRCA2* carriers are luminal-like, a similar proportion as in the general population of breast cancer patients^4^.

In the Icelandic population, there is one predominant pathogenic *BRCA2* mutation, 999del5 (rs80359671, NM_000059.3:c.767_771delCAAAT, NP_000050.2:p.Asn257LysfsTer17)^5,6^. It is carried by 0.8% of the general population^7^ and by an estimated 7–8% of breast cancer patients^7,8^. Other pathogenic *BRCA1* and *BRCA2* variants are very rare^9^, which confers an advantage when screening for and studying the nature of BRCA2-associated cancer in the Icelandic population.

In 2013, we reported an adverse outcome associated with a positive ER status for breast cancer patients carrying this mutation, whereas the traditional superior outcome was observed for non-carriers^10^. This was confirmed in a larger group of Icelandic carriers^11^. Similar findings have since been reported from other populations including a wide range of pathogenic *BRCA2* variants^12–18^. The notion that ER+ cancer is of a different nature in *BRCA2* mutation carriers than in sporadic cases is further supported by findings of higher Oncotype DX Breast Recurrence Scores for *BRCA*-associated versus sporadic ER+ cancer^19–21^ and by the observation that mortality among ER+ *BRCA2* carriers is higher than expected by the PREDICT model^22^.

Young women with breast cancer constitute another specific group of breast cancer patients for whom a positive ER status is not associated with superior survival^23–26^ and the survival disadvantage appears to be mainly restricted to the Luminal A type^23,24^. To what extent this observation of inferior prognosis in young breast cancer patients can be explained by the relatively high prevalence of *BRCA1/2* mutations among young patients^6,8,27,28^ is currently not known.

The specific nature and clinical presentation of ER+ tumors in *BRCA2* carriers and in patients diagnosed 40 years or younger has biological and clinical implications and needs to be better understood. We used nationwide data over a 24-year period from Icelandic population-based registries and studied nodal involvement, tumor size, tumor grade, and survival, according to ER status in those two groups compared with patients who were older than 40 years at diagnosis. The setting is unique as 85% of all breast cancer patients diagnosed during the study period had been tested for the virtually single pathogenic *BRCA1/2* mutation in the population. We could thus study and compare survival, clinical parameters, and treatment according to ER status in *BRCA2*-carriers, young (≤40 years) non-carriers, and older (>40 years) non-carriers.

## Results

**Fig. 1:**
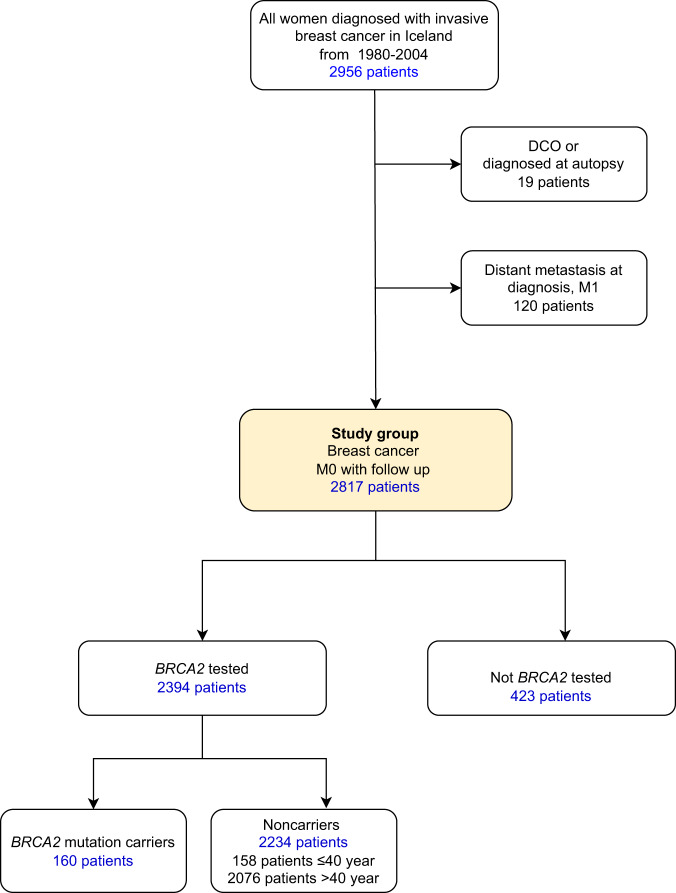
Flow diagram of the study group, showing exclusions and the number of patients according to *BRCA2* mutationstatus and age at diagnosis. DCO diagnosis was based on death certificate only, M1 distant metastases present at diagnosis, M0 no known metastases at diagnosis, *BRCA2* the Icelandic *BRCA2* founder mutation 999del5.

A total of 2956 women were diagnosed with breast cancer in the period 1980–2004. After excluding patients who were registered on the basis of death certificate only (DCO) or autopsy (19 patients) and patients with distant metastases (120 patients), 2817 patients remained. Of them, 2394 (85.0%) had been tested for the *BRCA2* mutation in earlier studies. A total of 160 women (6.7% of genotyped) carried the 999del5 *BRCA2* mutation (Fig. 1). Prevalence of the *BRCA2* mutation among tested patients ≤40 years of age was 21.0%, but it was 5.4% among women diagnosed older than 40 years of age.

After identifying the 160 *BRCA2*-carriers, the remaining 2234 non-carriers were divided into 158 young and 2076 older patients. Table 1 presents tumor characteristics for the three mutually exclusive groups. *BRCA2* carriers and young patients had larger average tumor sizes and a higher proportion of positive lymph nodes, tumor grade 3, and high Ki-67 than the older patients. Young cases had the lowest proportion of ER+ tumors (60.7%) but the highest proportion of HER2-positive tumors (27.9%). *BRCA2* carriers had a low proportion of HER2-positive tumors (3.4%).

**Table 1:** Clinical characteristics and treatment of patients.

	*BRCA2* 999del5 carrier cases	Non-carriers ≤40 years old	Non-carriers >40 years old
Total no.	160	158	2076
*Year of diagnosis, median [range]*	1992 [1980–2004]	1991 [1980–2004]	1994 [1980–2004]
*Age at diagnosis, median [range]*	47 [29–77]	36 [25–40]	60 [41–97]
*Deaths, no. (% of all cases)*	114 (71.3)	84 (53.2)	1388 (66.9)
BC deaths, no. (% of all cases)	72 (45.0)	68 (43.0)	561 (27.0)
*Tumor size, average [range] mm*	27.0 [5–110]	26.4 [3–100]	22.2 [0–150]
Unknown size	13	12	91
T1, no. (%)	75 (51.0)	70 (48.0)	1190 (59.8)
T2, no. (%)	57 (38.8)	66 (45.2)	686 (34.5)
T3, no. (%)	13 (8.8)	10 (6.9)	92 (4.6)
T4, no. (%)	2 (1.4)	0 (0.0)	21 (1.1)
Unknown	13	12	87
*Lymph node, no. (%)*			
N0	75 (48.1)	72 (46.5)	1090 (58.8)
N+	81 (51.9)	83 (53.6)	763 (41.2)
Unknown	4	3	223
*Grade, no. (%)*			
1	20 (12.8)	25 (21.6)	349 (26.5)
2	73 (46.8)	44 (37.9)	583 (44.2)
3	63 (40.4)	47 (40.5)	387 (29.3)
Unknown	4	42	757
*ER, no. (%)*			
Positive	113 (71.5)	85 (60.7)	1295 (74.6)
Negative	45 (28.5)	55 (39.3)	441 (25.4)
Unknown	2	18	340
*PR, no. (%)*			
Positive	96 (61.2)	82(60.3)	1067 (63.3)
Negative	61 (38.8)	54 (39.7)	619 (36.7)
Unknown	3	22	390
*HER2, no. (%)*			
Positive	5 (3.4)	24 (27.9)	76 (15.5)
Negative	143 (96.6)	62 (72.1)	413 (84.5)
Unknown	12	72	1587
*Ki-67, no. (%)*			
High	87 (59.2)	55 (67.9)	180 (43.6)
Low	60 (40.8)	26 (32.1)	233 (56.4)
Unknown	13	77	1663
*Molecular subtypes, no. (%)*			
Luminal A-like	37 (26.8)	20 (26.3)	197 (43.0)
Luminal B-like	68 (49.3)	27 (35.5)	145 (31.7)
Luminal B-like HER2−	64 (46.4)	14 (18.4)	113 (24.7)
Luminal B-like HER2+	4 (2.9)	13 (17.1)	32 (7.0)
Non-luminal HER2+-like	1 (0.7)	7 (9.2)	40 (8.7)
Triple negative (ductal)	32 (23.2)	22 (29.0)	76 (16.6)
Unknown	22	82	1618
*Surgery at diagnosis*			
Lumpectomy	38 (23.8)	54 (34.6)	738 (37.8)
Mastectomy	122 (76.3)	102 (65.4)	1216 (62.2)
Unknown	0	2	122
*Adjuvant chemotherapy*			
None	73 (45.9)	58 (37.4)	1334 (69.0)
Any	86 (54.1)	97 (62.6)	598 (31.0)
Unknown	1	3	144
*Adjuvant hormone therapy*			
None	92 (57.9)	110 (71.9)	1008 (53.4)
Any	67 (42.1)	43 (28.1)	881 (46.6)
Unknown	1	5	187

Non-carriers: Patients who tested negative for the 999del5 *BRCA2* mutation.

*ER* estrogen receptor, *PR* progesterone receptor.

### Tumor characteristics and treatment according to ER status

For ER+ cancer, lymph node metastases were present in 60.9%, 54.8%, and 42.5% of *BRCA2*-carriers, young and older patients, respectively, whereas for ER-negative cancer the proportion with lymph node involvement was lowest in *BRCA2*-carriers and highest in young patients (Table 2b). Similarly for ER+ cancer, tumors were largest in *BRCA2*-carriers, and smallest in older patients, whereas for ER-negative cancer the largest tumors were seen in young patients (Table 2a). In the ER-negative stratum, the proportion with grade 3 was much higher than in the ER+ stratum for all three patient groups (Table 2c). However, in the ER+ stratum, *BRCA2*-carriers had a higher proportion of tumor grade 3 than the other two groups. Around and over 60% of the patients had mastectomies. The highest proportion was among *BRCA2* carriers, both for ER+ (77.9%) and ER-negative (71.1%) patients. Among cases with ER+ tumors, only around 30% received chemotherapy in the older group, but around 60% in the two other groups (Tables 1 and 2).

**Table 2:** *BRCA2* carriers and young non-carrier patients compared with older non-carrier patients stratified by ER status.

		*BRCA2* 999del5 carrier patients	Non-carrier patients ≤40 years old	Non-carrier patients >40 years old
	Total number with known ER status	158	140	1736
a	*Average tumor size, mm*			
	ER-positive	28.6 [6–110]	24.5 [3–90]	21.7 [0–150]
	ER-negative	23.3 [5–65]	29.1 [5–100]	25.3 [2–120]
	Unknown size	12	6	39
b	*Lymph node, no. (%)*			
	ER-positive			
	N0	43 (39.1)	38 (45.2)	671 (57.5)
	N+	67 (60.9)	46 (54.8)	496 (42.5)
	ER-negative			
	N0	32 (72.7)	24 (44.4)	230 (56.1)
	N+	12 (27.3)	30 (55.6)	180 (43.9)
	Unknown nodal status	4	2	159
c	*Grade, no. (%)*			
	ER-positive			
	1	15 (13.6)	22 (32.8)	261 (30.1)
	2	58 (52.7)	29 (43.3)	434 (50.1)
	3	37 (33.6)	16 (23.9)	172 (19.8)
	ER-negative			
	1	4 (8.9)	2 (4.4)	37 (11.8)
	2	15 (33.3)	15 (32.6)	99 (31.4)
	3	26 (57.8)	29 (63.0)	179 (56.8)
	Unknown grade	3	27	554
d	*Treatment, no. (%)*			
	ER-positive			
	Mastectomy	88 (77.9)	56 (65.9)	767 (61.0)
	Chemotherapy	65 (57.5)	54 (63.5)	370 (29.8)
	Hormone therapy	58 (51.3)	33 (38.8)	749 (62.2)
	ER-negative			
	Mastectomy	32 (71.1)	32 (59.3)	266 (63.8)
	Chemotherapy	20 (44.4)	36 (66.7)	176 (42.8)
	Hormone therapy	8 (17.8)	9 (17.3)	85 (20.7)

^a^Mean tumor size.

^b^Nodal involvement.

^c^Tumor grade distribution.

^d^Treatment.

Non-carriers: Patients who tested negative for the 999del5 BRCA2 mutation.

*ER* estrogen receptor.

### Survival according to ER status and treatment

**Fig. 2:**
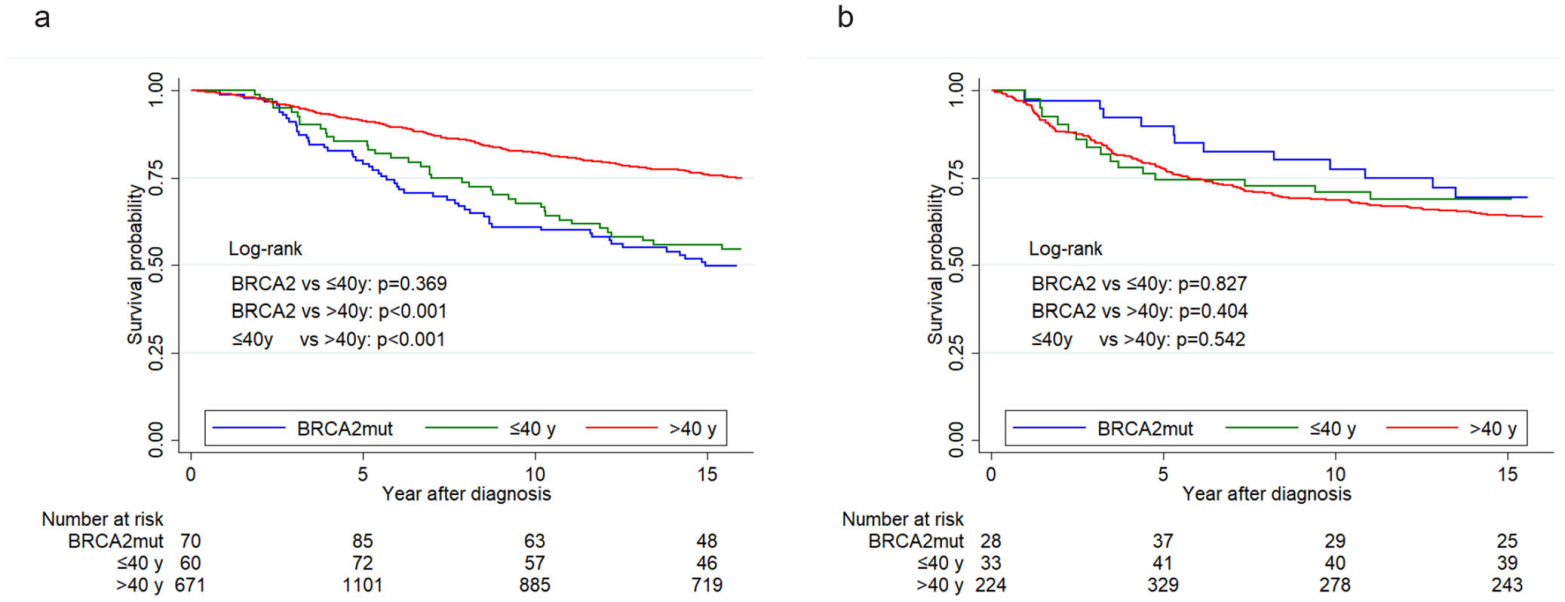
Breast cancer-specific survival according to ER status. **a** ER-positive cancer. **b** ER-negative cancer. ER estrogen receptor. BRCA2 carriers of the Icelandic founder mutation 999del5.

The median follow-up time was 15.5 years. Figure 2a shows that for ER+ cancer 15-year survival differed significantly between the older group on one hand (76.0%; 95% CI: 73.4–78.4), and *BRCA2*-carriers (49.7%; 95% CI: 39.7–59.0) and the young group (55.8%; 95% CI: 44.6–65.7) on the other hand. The picture was different for ER-negative cancer (Fig. 2b), where 15-year survival did not differ significantly between any of the three groups and ranged between 64% and 69.3%. Adjusting for year and age at diagnosis the hazard ratio comparing ER+ and ER-negative tumors was for *BRCA2* carriers 2.06 (95% CI: 1.11–3.83); for young patients, it was 1.66 (95% CI: 0.94–2.94), whereas for older patients it was 0.64 (95% CI: 0.52–0.78) (Table 3a). When adjusted for other clinical variables HRs were not statistically significantly different from 1.00 (Table 3b).

**Table 3:** Risk of breast cancer-specific death according to ER status, other prognostic factors and patient group.

		*BRCA2* mutation carrier patients	Non-carrier patients ≤40 years old	Non-carrier patients >40 years old
		HR (95% CI)	HR (95% CI)	HR (95% CI)
	Total number with known ER status	158	140	1736
a^1)^	ER-negative	1.00	1.00	1.00
	ER-positive	2.06 [1.11–3.83]	1.66 [0.94–2.94]	0.64 [0.52–0.78]
b^2)^	ER-negative	1.00	1.00	1.00
	ER-positive	1.49 [0.72–3.10]	1.65 [0.73–3.73]	1.00 [0.73–1.37]
	Size (continuous variable)	1.03 [1.01–1.04]	1.03 [1.01–1.05]	1.02 [1.01–1.03]
	Lymph node N0	1.00	1.00	1.00
	Lymph node N+	2.97 [1.20–7.36]	3.64 [1.08–12]	2.95 [2.08–4.18]
	Grade 1	1.00	1.00	1.00
	Grade 2	0.57 [0.25–1.29]	1.19 [0.53–2.69]	2.33 [1.50–3.61]
	Grade 3	0.50 [0.21–1.15]	0.52 [0.20–1.39)	3.08 [1.94–4.90]
	Lumpectomy	1.00	1.00	1.00
	Mastectomy	0.50 [0.24–1.03]	0.90 [0.40–2.05]	1.49 [1.07–2.07]
	No adjuvant chemotherapy	1.00	1.00	1.00
	Adjuvant chemotherapy	0.50 [0.23–1.12]	0.70 [0.19–2.50]	0.82 [0.58–1.16]
	No adjuvant hormone therapy	1.00	1.00	1.00
	Adjuvant hormone therapy	0.95 [0.38–2.39]	1.02 [0.46–2.27]	0.82 [0.59–1.14]
	Missing information (*n*)	19	38	730
c^3)^	Triple negative (ductal)	1.00	1.00	1.00
	Luminal A-like	2.85 [1.07–7.57]	2.30 [0.78–6.82]	0.62 [0.37–1.04]
	Luminal B-like	3.47 [1.42–8.49]	2.92 [1.04–8.18]	1.20 [0.76–2.03]
	Non-luminal HER2+-like	–	1.46 [0.27–7.87]	1.89 [1.03–3.45]
	Missing cases (*n*)	20	64	1278

Multivariate Cox proportional hazards model including*: ^a^ER status.

^ b^Tumor size (continuous), nodal involvement, tumor grade, and treatment.

^ c^Intrinsic-like subtypes.

Non-carriers: Patients who tested negative for the 999del5 *BRCA2* mutation.

*CI* confidence interval, *ER* estrogen receptor.

*Year at diagnosis and age at diagnosis included in all three models.

1) Interaction test for association between survival and:

ER status, comparing *BRCA2* mutation carriers and >40 years old non-carriers, *P*-value < 0.001.

ER status, comparing ≤40 years old non-carriers and >40 years old non-carriers, *P*-value = 0.005.

2) Interaction test for association between survival and:

Grade 3, comparing *BRCA2* mutation carriers and >40 years old non-carriers, *P*-value < 0.001.

Grade 3, comparing ≤40 years old non-carriers and >40 years old non-carriers, *P*-value < 0.001.

Mastectomy, comparing *BRCA2* mutation carriers and ≤40 years old non-carriers, *P*-value = 0.010.

Mastectomy, comparing *BRCA2* mutation carriers and >40 years old non-carriers, *P*-value = 0.001.

Chemotherapy, comparing *BRCA2* mutation carriers and ≤40 years old non-carriers, *P*-value = 0.040.

3) Interaction test for association between survival and:

Intrinsic-like subtype Luminal A comparing *BRCA2* mutation carriers and >40-year-old non-carriers, *P*-value = 0.003.

Intrinsic-like subtype Luminal A comparing ≤40-year-old non-carriers and >40-years-old non-carriers, *P*-value = 0.039.

Intrinsic-like subtype Luminal B comparing *BRCA2* mutation carriers and >40-year-old non-carriers, *P*-value = 0.035.

Tumor grade 3 predicted superior survival compared with grade 1 in *BRCA2* carriers and young non-carriers, in contrast with the over threefold risk of death associated with grade 3 that was seen in the older group.

For patients with information allowing definition of intrinsic-like subtypes (Table 3c), Luminal A-like cancer was associated with an increased risk of death among *BRCA2* carriers and young non-carriers, contrasting with the lower risk among the older group.

Interaction tests (Table 3) indicated that ER status, Luminal A-like subtype, and grade predicted prognosis similarly for *BRCA2* carriers and young cases, but differently than for older cases. For the Luminal B-like subtype, the association with prognosis differed only between *BRCA2* carriers and older cases, here the young cases were not distinct from the older group. Mastectomy conferred significantly stronger protection for *BRCA2* carriers than for either young or older cases, and for chemotherapy, a significant distinction was present between carriers and young cases.

### Patients not tested for the BRCA2 999del5 founder mutation

The 15% of all female breast cancer patients diagnosed in 1980–2004 who were not tested (423 cases), were older at diagnosis (median age 72 years versus 58 years) and they more often lacked histological confirmation for diagnosis (13.2%) than the genotyped group (0.2%).

## Discussion

Opposite prognostic effects associated with ER status and proliferation in *BRCA2* carriers, as compared with non-carriers, were first reported in Icelandic studies^10,11^ and have since been confirmed in several other populations^12–18^. Young breast cancer patients have also been found to have an opposite prognostic association with ER status as compared with older patients^23–26^.

Based on unique population-based data from Iceland where 85% of patients had been tested for the Icelandic *BRCA2* founder mutation we show here that young women with breast cancer shared the abovementioned unconventional prognostic associations with *BRCA2* carriers even though virtually no *BRCA* mutation carriers were included in the young group, rendering it unlikely that the worse outcomes associated with an ER+ status could be explained by the high prevalence of germline *BRCA* mutations in young breast cancer patients. Alternative explanations might be related to epigenetic changes in *BRCA* genes, or other disturbances in biological processes rather than a lack of BRCA2 protein, perhaps due to other inherited mutations in those young women.

*BRCA2*-carriers and young non-carriers with ER+ cancer had considerably lower 15-year survival than older non-carriers, contrasting with ER-negative cancer where survival did not differ significantly between the three groups. Similarly, survival was significantly worse if tumors were ER+ than ER-negative for *BRCA2* carriers and non-significantly worse for young patients, whereas ER+ cancer was associated with good prognosis among older patients as expected.

Interaction tests supported the reverse associations between survival and ER status, Luminal A-like subtype, and grade for both *BRCA2*-carriers and young patients as compared with older cases. They also indicated that mastectomy conferred stronger protection for *BRCA2* carriers than for either young or older cases and that chemotherapy had a stronger effect in carriers than in young cases.

In addition to the abovementioned similarities in outcomes, both *BRCA2* carriers and young patients had more advanced disease at diagnosis than the group of older patients. However, the two groups differed in that only for *BRCA2* carriers was a positive ER status associated with positive lymph nodes and larger tumors. Furthermore, the young cases had a relatively low proportion of ER+ tumors and a high proportion of HER2-positive tumors compared with only 3.4% among *BRCA2* carriers. The high prevalence of HER2-positive tumors in the young patient group, or 28%, must have affected their survival in a negative fashion, as all patients in the study were diagnosed before the general uptake of HER2-targeted drugs. However, this cannot explain the reverse prognostic effect of an ER+ status in the young group, because the proportion of HER2-positive tumors in this group was the same without respect to whether tumors were ER+ or ER-negative.

The 21-gene expression assay Oncotype DX Breast Recurrence Scores^®^ is one example of scores that quantify the likelihood of breast cancer recurrence and the potential benefit of chemotherapy in ER+ patients. The notion that ER+ breast cancer in *BRCA2* carriers has special prognostic properties is supported by findings of increased rate of high recurrence scores (RS) in *BRCA1/2* carriers^19–21^. For example, Halpern et al. found an approximately 3-fold increased rate of high RS among *BRCA1/2* carriers with ER+ breast cancer in comparison with a large group of patients from the general population and concluded that this “might indicate that hormone-positive breast cancers in *BRCA* carriers are molecularly unique”^20^. Along the same line, it was recently reported that the observed mortality among *BRCA2* carriers was higher than expected according to the PREDICT model^22^. Thus, there are indications that *BRCA2* carriers with ER+ cancer would benefit more from chemotherapy than non-carriers with ER+ cancer. Results from a Nordic study are also interesting in this context, where the majority of *BRCA2*-mutated tumors belonged to an unusual genomic subgroup of Luminal A-classified tumors, a so-called luminal-complex group defined by global DNA copy number and gene-expression profiling. This subgroup had relatively poor survival^29^. Finally, *BRCA2* mutated tumors have a particularly high degree of methylation compared to *BRCA1* mutated tumors^30^, emphasizing the distinction of *BRCA2* mutated tumors.

Interestingly, high tumor grade did neither correlate with a worse prognosis in young breast cancer patients nor in *BRCA2* mutation carriers. A similar lack of predictive potential for grade among *BRCA2* carriers has been reported before^13,15,22^ and it could be related to the reverse association with proliferation^10^ because evaluation criteria for histological grade are highly influenced by proliferation. However, in all three groups, the conventional association between ER+ status and lower grades was present. It is noteworthy that the inferior prognosis associated with ER+ breast cancer among *BRCA2* mutation carriers cannot be explained by lack of response to antihormone therapy because the prognostic disadvantage was found to be similar for *BRCA2* carriers diagnosed during the period of 1935–1979 before antihormonal therapy was available, as it was for patients diagnosed during 1980–2012^11^. Therefore, it is likely to be due to some specific nature of breast tumors in *BRCA2* mutation carriers.

This population-based study is unique as 85% of all breast cancer cases diagnosed in the period of 1980–2004 in Iceland were tested for the *999del5 BRCA2* founder mutation. Therefore, it provides the unprecedented opportunity to study young breast cancer cases who do not carry this founder mutation, and in effect, virtually no *BRCA1/2* mutations, because of the very low prevalence of other *BRCA1/2* mutations in the population. The 15% of the patients who were not screened for the mutation were considerably older than the 85% tested, which renders it likely that most mutation carriers diagnosed in 1980–2004 were included in this study. Survival bias is not likely to be a problem because for 51% of the cases, paraffin-embedded tissue removed at surgery was used for *BRCA2*-mutation testing, and for most of the remaining women diagnostic blood samples were used, or samples collected shortly after diagnosis in context with research on familial breast cancer during the nineties. Only around 10% of the patients had *BRCA2* testing done >2 years from diagnosis, and the Left-truncated survival analysis should minimize the resulting potential bias. Finally, usage of the unique civil personal registration number rendered the record linkage accurate and the follow-up complete. Although only one pathogenic *BRCA2* variant was studied here, the results are in accordance with other studies reporting on similar survival patterns for other pathogenic *BRCA2* variants^12–18^.

## Conclusion

This study confirms previously published results of unfavorable breast cancer prognosis associated with ER+ tumors, especially Luminal A-like tumors, in young women as well as *BRCA2*-carriers. The results further indicate that those characteristics among young patients cannot be explained by the high prevalence of *BRCA1/2* mutation carriers in this patient group. Those two clinically important patient groups need special attention with respect to treatment choices, in particular, if diagnosed with ER+ tumors. Clinical Gene Expression-based Assays may be of special value to them. *BRCA2* carriers appear to benefit more from mastectomy and chemotherapy than do non-carriers. It is thus advisable to have knowledge of *BRCA2* status when treatment decisions are made. Finally, it is important to understand the biological basis for the specific nature of ER+ tumors in young women and *BRCA2*-carriers.

## Methods

### Setting

We conducted this study in Iceland, where a unique civil personal registration number is assigned to all citizens at birth or immigration, enabling individual-level data linkage across all registries including the nationwide Icelandic Cancer Registry, the Cause of Death Registry, and clinical databases at Icelandic Hospitals. All citizens have access to mainly tax-funded health care at public hospitals and all cancer patients are treated at either of the two public hospitals. The presence of a single *BRCA2* founder mutation and a very low prevalence of other *BRCA1-* and *2* mutations in the population^9^ facilitate screening for *BRCA* mutations when studying the clinical presentation and prognosis in *BRCA2* mutation carriers.

### Ethics

The study was approved by the Icelandic Science Ethical Committee (SEC) (VSN-13-133) and was conducted in accordance with the General Data Protection Regulation (GDPR), whereupon all women who were alive at the time of study gave their written informed consent, and SEC allowed usage of paraffin blocks and other relevant information for those who were deceased. Authors complied with all relevant ethical regulations including the Declaration of Helsinki.

### Study population

The study cohort included all (2956) Icelandic women diagnosed with invasive breast cancer in Iceland from the year 1980 through the year 2004 (Fig. 1), based on the nationwide, high-quality Icelandic Cancer Registry (ICR)^31^. Therefore 19 were excluded from this study because the diagnosis was based on autopsy and death certificate-only (DCO) and 120 cases were excluded as they had distant metastases at diagnosis, leaving 2817 patients for analysis. Thereof 2394 patients (85%) had been tested for the *BRCA2* 999del5 founder pathogenic variant in the period 1995–2012 in the context of research projects. The majority had been selected for *BRCA2* testing according to defined periods of diagnosis and year of birth, see further description in Jonasson et al.^11^. We compared the basis of diagnosis, age at diagnosis, tumor size, nodal status, grade and 15-year survival among tested patients, divided into three groups: Carriers of the 999del5 *BRCA2* pathogenic variant, young (≤40 years at diagnosis) non-carriers, and older (older than 40 years at diagnosis) non-carriers.

### Prognostic factors and treatment

Routine assessment of pathological parameters for breast cancer in Iceland is centrally performed at the Pathology Departments of Landspitali University Hospital and Akureyri Hospital. For ER and PR status, routine assessment was initiated in 1981 using Dextran-coated charcoal assay until 1995, succeeded by immunohistochemical (IHC) staining. Tumors are considered positive for the estrogen- and progesterone receptors when >1% of cell nuclei stain positive for the receptors with immunohistochemistry. Routine assessment of Ki-67 protein status started in 2007, with the cut-off point for high staining being at 14%. HER2 expression status has been assessed since 2004 using IHC and applying fluorescence in situ hybridization if results are borderline.

For *BRCA2* mutation carriers with missing pathological information plus two non-carrier controls individually matched on year of birth and diagnosis, Tissue Micro Arrays were constructed from paraffin blocks when available, and missing values for pathological variables were assessed using IHC^11^.

We used the following definitions for intrinsic-like subtypes: ‘*Luminal A-like*’: (ER+/PR+/HER2− and low Ki-67 (grade 1 and 2 for missing Ki-67)), ‘*Luminal B-like (HER2 negative)*’: (ER+/HER2−, PR− and/or high Ki-67 (or grade 3 for missing Ki-67)), ‘*Luminal B-like (HER2 positive)*’: (ER+/HER2+ with any PR and Ki-67 status), ‘*HER2 positive (non-luminal)*’: (ER−/PR−/HER2+), ‘*Triple-negative (basal-like)*’: (ER−/PR−/HER2−). We had to combine Luminal B-like HER2 negative and HER2 positive for the survival analysis because there were only 4 *BRCA2* carriers who were HER2 positive.

Information on treatment was abstracted from patient charts, the date of death was ascertained by record linkage with Statistics Iceland, and the cause of death by record linkage with Statistics Iceland and the Directorate of Health. Data were obtained blinded with respect to mutation status.

### Statistical analysis

Mean values for continuous variables were compared between *BRCA2* mutation carrier cases and the remainder of patients from the general population stratified into two groups according to age at diagnosis (40 years or less (young) and older patients), using the t-test statistic. The *χ*^2^-test was used for comparing proportions.

Univariable survival curves were generated using Kaplan–Meier methods and the log-rank test was used for estimating *P*-values. Hazard ratios (HRs) were estimated using the Cox proportional hazard model. Multivariable analyses were conducted by first including only ER status or intrinsic-like subtypes, year of birth, and year of diagnosis, and thereafter the covariates tumor size, nodal involvement, tumor grade, and treatment (mastectomy, chemotherapy, hormone treatment). Follow-up for breast cancer-specific survival was from the date of diagnosis of the first invasive breast cancer until death or the last date of follow-up (December 31st, 2020). Patients who died of other causes than breast cancer were censored at the date of death. Of the women included in this study, 51% had been tested for the *BRCA2* founder mutation by using a paraffin-embedded tumor specimen and 49% by using blood samples, and for the majority of those, blood had been drawn either before diagnosis, at diagnosis or within 2 years from diagnosis. Left-truncated survival analysis^32^ was used to avoid survivorship bias with follow-up time starting on the date of sampling for patients tested after diagnosis. Using the Cox model we tested for interaction between ER status, grade, and intrinsic-like subgroup on one hand and group (*BRCA2*-carriers, young non-carriers, or older non-carriers) on the other hand, in separate models by including a multiplication factor between each set of variables.

Statistical tests were two-sided and a *P*-value of <0.05 was considered statistically significant. All analyses were performed using the STATA Statistical Software Stata/IC 14.0 for Windows.

### Supplementary Information

Related Manuscript File

## Data Availability

The study participant phenotype data are not publicly available due to the protection of participant privacy and confidentiality. Data sharing is not possible under the Icelandic Science Ethical Committee (SEC) constraints. However, data can be made available in an anonymized form upon a reasonable request and after approval from the SEC.

## Code availability

Analysis codes are available from the corresponding author upon reasonable request.
