# Supplementary material for: Estrogen receptor-positive breast cancer and adverse outcome in BRCA2 mutation carriers and young non-carrier patients
Source: NPJ Breast Cancer. 2023 Nov 30;9:95. doi: 10.1038/s41523-023-00600-8 (PMC10689749; doi:10.1038/s41523-023-00600-8)
Supplement: Supplementary file 1 — Related Manuscript File [file 41523_2023_600_MOESM1_ESM.pdf]

nature portfolio

Corresponding author(s): Laufey Tryggvadottir

Last updated by author(s): Sep 11, 2023

Reporting Summary

Nature Portfolio wishes to improve the reproducibility of the work that we publish. This form provides structure for consistency and transparency in reporting. For further information on Nature Portfolio policies, see our Editorial Policies and the Editorial Policy Checklist.

Statistics

For all statistical analyses, confirm that the following items are present in the figure legend, table legend, main text, or Methods section.

n/a	Confirmed	
☐	☒	The exact sample size (*n*) for each experimental group/condition, given as a discrete number and unit of measurement
☒	☐	A statement on whether measurements were taken from distinct samples or whether the same sample was measured repeatedly
☐	☒	The statistical test(s) used AND whether they are one- or two-sided<br>*Only common tests should be described solely by name; describe more complex techniques in the Methods section.*
☐	☒	A description of all covariates tested
☒	☐	A description of any assumptions or corrections, such as tests of normality and adjustment for multiple comparisons
☐	☒	A full description of the statistical parameters including central tendency (e.g. means) or other basic estimates (e.g. regression coefficient) AND variation (e.g. standard deviation) or associated estimates of uncertainty (e.g. confidence intervals)
☐	☒	For null hypothesis testing, the test statistic (e.g. *F*, *t*, *r*) with confidence intervals, effect sizes, degrees of freedom and *P* value noted<br>*Give P values as exact values whenever suitable.*
☒	☐	For Bayesian analysis, information on the choice of priors and Markov chain Monte Carlo settings
☒	☐	For hierarchical and complex designs, identification of the appropriate level for tests and full reporting of outcomes
☒	☐	Estimates of effect sizes (e.g. Cohen's *d*, Pearson's *r*), indicating how they were calculated

Our web collection on statistics for biologists contains articles on many of the points above.

Software and code

Policy information about availability of computer code

Data collection	*Provide a description of all commercial, open source and custom code used to collect the data in this study, specifying the version used OR state that no software was used.*
Data analysis	*Provide a description of all commercial, open source and custom code used to analyse the data in this study, specifying the version used OR state that no software was used.*

For manuscripts utilizing custom algorithms or software that are central to the research but not yet described in published literature, software must be made available to editors and reviewers. We strongly encourage code deposition in a community repository (e.g. GitHub). See the Nature Portfolio guidelines for submitting code & software for further information.

Data

Policy information about availability of data

All manuscripts must include a data availability statement. This statement should provide the following information, where applicable:

- Accession codes, unique identifiers, or web links for publicly available datasets
- A description of any restrictions on data availability
- For clinical datasets or third party data, please ensure that the statement adheres to our policy

The study participant phenotype data are not publicly available due to protection of participant privacy and confidentiality. Data sharing is not possible under the

Icelandic Science Ethical Committee (SEC) constraints. But data can be made available in an anonymized form upon a reasonable request and after approval from the SEC

Research involving human participants, their data, or biological material

Policy information about studies with human participants or human data. See also policy information about sex, gender (identity/presentation), and sexual orientation and race, ethnicity and racism.

Reporting on sex and gender	This study concerns women with breast cancer
Reporting on race, ethnicity, or other socially relevant groupings	No socially relevant grouping was used, the study included all women diagnosed in Iceland during a specified period
Population characteristics	The study included all women diagnosed in Iceland during a specified period
Recruitment	The Icelandic Cancer Registry was the study base
Ethics oversight	Icelandic Science Ethical Committee

Note that full information on the approval of the study protocol must also be provided in the manuscript.

Field-specific reporting

Please select the one below that is the best fit for your research. If you are not sure, read the appropriate sections before making your selection.

☒ Life sciences ☐ Behavioural & social sciences ☐ Ecological, evolutionary & environmental sciences

For a reference copy of the document with all sections, see nature.com/documents/nr-reporting-summary-flat.pdf

Life sciences study design

All studies must disclose on these points even when the disclosure is negative.

Sample size	All eligible women (see exclusions below) with breast cancer diagnosed in Iceland during 1980-2004 formed the study group, a total of 2817 women
Data exclusions	The study cohort included all (2956) Icelandic women diagnosed with invasive breast cancer in Iceland from year 1980 through year 2004 based on the nationwide Icelandic Cancer Registry. Thereof 19 were excluded from this study because diagnosis was based on autopsy or death certificate-only and 120 cases were excluded as they had distant metastases at diagnosis, leaving 2817 patients for analysis.
Replication	Not applicable
Randomization	Not applicable
Blinding	Retrieval of clinical information for the participants was done without knowledge of their BRCA2-mutational status

Reporting for specific materials, systems and methods

We require information from authors about some types of materials, experimental systems and methods used in many studies. Here, indicate whether each material, system or method listed is relevant to your study. If you are not sure if a list item applies to your research, read the appropriate section before selecting a response.

Materials & experimental systems

n/a	Involved in the study	
☒	☐	Antibodies
☒	☐	Eukaryotic cell lines
☒	☐	Palaeontology and archaeology
☒	☐	Animals and other organisms
☐	☒	Clinical data
☒	☐	Dual use research of concern
☒	☐	Plants

Methods

n/a	Involved in the study	
☒	☐	ChIP-seq
☒	☐	Flow cytometry
☒	☐	MRI-based neuroimaging

Clinical data

Policy information about clinical studies

All manuscripts should comply with the ICMJE guidelines for publication of clinical research and a completed CONSORT checklist must be included with all submissions.

Clinical trial registration	This was not a clinical trial. Information on pathological parameters came from the Icelandic Cancer Registry, treatment was abstracted from patient charts, date of death was ascertained by record linkage with Statistics Iceland and cause of death by record linkage with Statistics Iceland and the Directorate of Health
Study protocol	Study protocol was approved by the Icelandic Science Ethical Committee
Data collection	Diagnosis period of participants was 1980-2004. Information on treatment was abstracted from patient charts, date of death was ascertained by record linkage with Statistics Iceland and cause of death by record linkage with Statistics Iceland and the Directorate of Health
Outcomes	Outcome measure was death from breast cancer, which was assessed by record linkage with information from Statistics Iceland and the Directorate of Health
